# Supplementary material for: Circulatory Neutrophils Exhibit Enhanced Neutrophil Extracellular Trap Formation in Early Puerperium: NETs at the Nexus of Thrombosis and Immunity
Source: Int J Mol Sci. 2021 Dec 20;22(24):13646. doi: 10.3390/ijms222413646 (PMC8704360; doi:10.3390/ijms222413646)
Supplement: Supplementary file 1 [file ijms-22-13646-s001.zip › ijms-1456388-supplementary.pdf]

Figure S1. Cell free nucleosomes and differential cell counts postpartum. (**a.**) In contrast to the serum levels, the plasma levels of cell free nucleosomes attained statistical significance. (**b.**) Polymorphonuclear neutrophil and mononuclear cell counts in peripheral blood from healthy donors, donors during pregnancy and 48 hours postpartum as determined by sandwich ELISA. P: pregnancy; PD: post-delivery. Data are presented as mean ± SEM. *P < 0.05, **P < 0.01, ***P < 0.001, ****P < 0.0001.

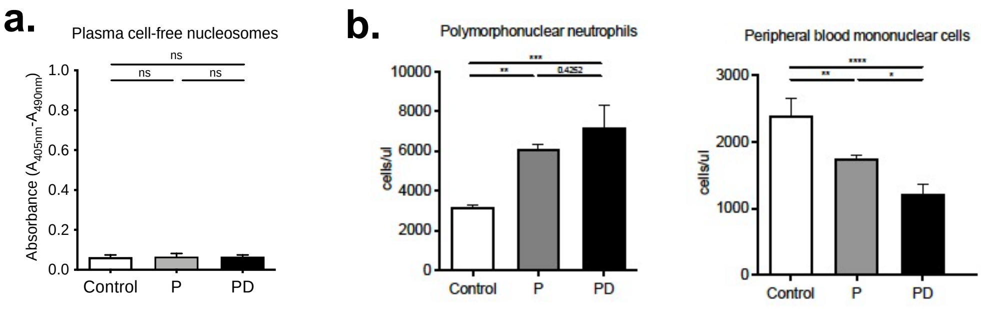

Figure S2. Oxidative burst and phagocytic activity evaluation of postpartum neutrophils. (**a.**) Intracellular oxidative burst in neutrophils from healthy control donors, donors during pregnancy and postpartum by DCFH-DA detection. (**b.**) Extracellular oxidative burst in neutrophils from healthy control donors, donors during pregnancy and postpartum by luminol assay. (**c.**) Phagocytic activity of neutrophils obtained from healthy female controls, donors during pregnancy and 48 hours post-delivery. P: pregnancy; PD: post-delivery; ecROS: extracellular ROS; icROS: intracellular ROS; RFU: relative fluorescence units; MLU: mean fluorescence units; AUC: area under the curve. Data are presented as mean ± SEM. *P < 0.05, **P < 0.01, ***P < 0.001, ****P < 0.0001. (one- or two-way ANOVA followed by Bonferroni's multiple comparison post-test). All experiments were performed at least 3 times with consistent results.

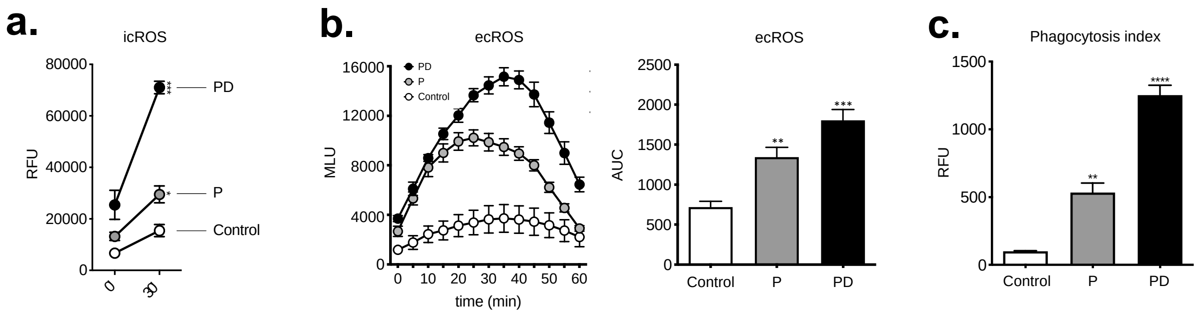

Figure S3. Serum and plasma concentrations of G-CSF in healthy non-pregnant controls, pregnant individuals and at post-delivery (**a.**). P: pregnancy; PD: post-delivery. Data are presented as mean ± SEM. **P < 0.01, ****P < 0.0001.

a.

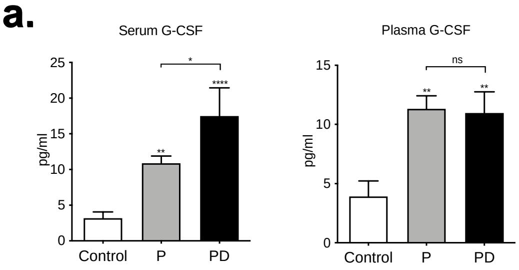

Figure S4. Quantitative and qualitative assessments of the utilized microparticles. (**a.**) Fluorescence histograms and bar graph depicting the quantitative analysis of microparticles in control, pregnancy and 48 hours postpartum plasma, positive for AnnexinV staining. Plasma was assessed for 60 seconds. P: pregnancy; PD: post-delivery. Data are presented as mean ± SEM. *P < 0.05, ***P < 0.001. (**b.**) Size, size distribution, concentration and volume determination of microparticles in control, pregnancy and 48 hours postpartum plasma.

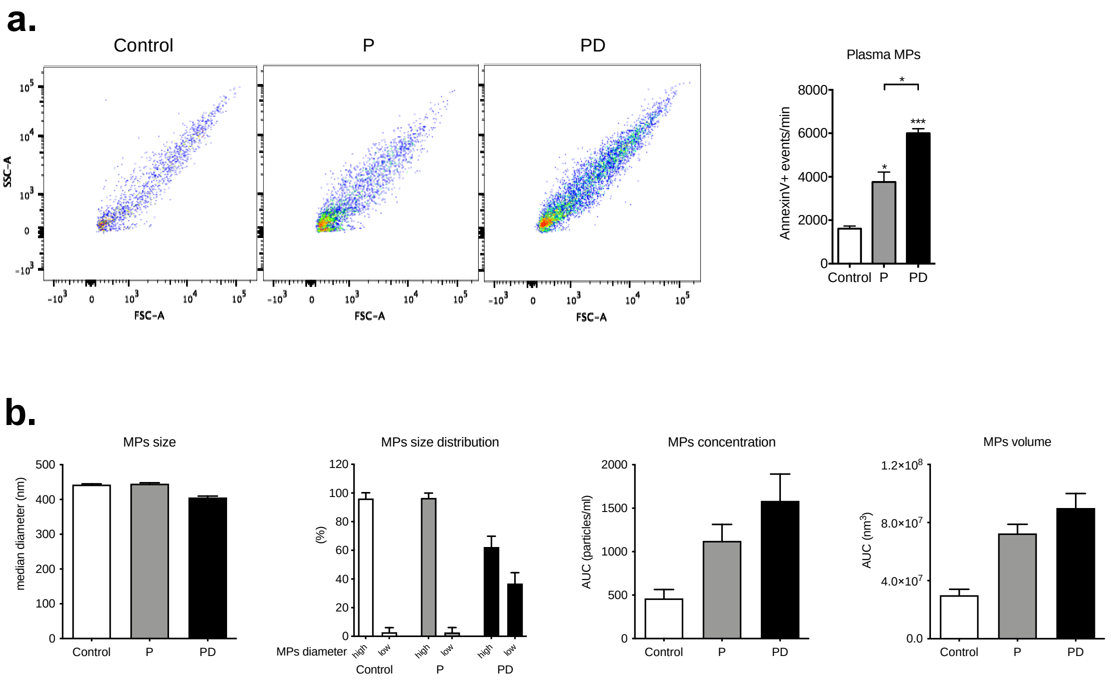

Table S1. Demographic characteristics of the study cohort, i.e. non-pregnant healthy controls, women during the three trimesters of pregnancy and women at postpartum.

	Non-pregnant	Pregnant	Postpartum	P
n	20	29	9	na
		IT: 5		
		IIT: 13		
		IIIT: 11		
Maternal age (years)	32 (23-46)	36 (26-41)	38 (25-40)	ns
Gestational age (weeks)	na	IT: 12 (12-13)	na	na
		IIT: 24 (22-26)		
		IIIT: 38 (37-41)		

Values expressed as mean ± SEM with minimum-maximum, where applicable; IT, first trimester; IIT, second trimester, IIIT, third trimester ns, not significant; na, not applicable.
